# Burnei’s Technique in the treatment of radial head displacement; innovative surgery. Study on two cases

**Published:** 2013-03-25

**Authors:** CA Ghinea, S Gavriliu, I Georgescu, C Vlad, E Japie, A Pârvan, R Ghiță

**Affiliations:** * Craiova County Emergency Hospital; ** “Carol Davila" University of Medicine and Pharmacy, Bucharest “M.S. Curie" Children’s Emergency Hospital, Bucharest; *** “M.S. Curie" Children’s Emergency Hospital, Bucharest; **** “Floreasca" Clinical Emergency Hospital Bucharest

**Keywords:** congenital radial head dislocation, posttraumatic radial head dislocation, ligament plasty, extensor carpi radialis longus

## Abstract

**Introduction:** Dislocation of the radial head, congenital or traumatic, anteriorly, posteriorly or laterally displaced, requires surgery to reseat and stabilize the head of the radius within the joint, in order to restore elbow flexion and, as much as possible, pronation-supination.

** Scope:** This article is meant to present the technique of proximal radial-ulnar ligament plasty using the extensor carpi radialis longus (ECRL) tendon, as well as other techniques for the stabilization of a dislocated radial head. The ECRL tendon technique, quadrate and annular ligament reconstruction variant was first used by Gh. Burnei in 1985, at Mangalia Municipal Hospital, Romania.

**Materials and method: ** This study contains two clinical cases, a 6-year-old girl with congenital dislocation of the radial head, and a 10-year-old boy with traumatic dislocation of the radial head, both of whom were treated by open reduction and stabilization of the dislocation with the ECRL tendon, using the Burnei procedure.

** Results:** In both cases, the operation was successful in the reduction and stabilization of the dislocated radial head, whose position was maintained in flexion-extension and pronation-supination, and in the reconstruction of the annular ligament using Burnei's procedure, variant 2.

Postoperatively, the clinical evolution was good, the patients having regained elbow mobility. The child with congenital dislocation exhibits normal flexion and pronation-supination within normal range, and the traumatic dislocation also exhibits normal flexion and pronation-supination limited with 20 degrees.

Radiologically, in both cases the radial head is anatomically placed relatively to the humeral capitellum, in both flexion and extension.

** Discussion:** Stabilization of the radial head in traumatic or congenital dislocation can only be surgically achieved. Congenital dislocation of the radial head requires the reconstruction of the proximal radial-ulnar joint, preferably at a young age, in order to avoid subsequent complications, culminating in ulnar or radial nerve paralysis. Traumatic dislocation of the radial head is usually accompanied by the fracture of the ulna, but may be encountered in isolation.

** Conclusions:** The Burnei procedure is an alternative for the treatment of radial head dislocation and is advantageous because of the use of a study, well vascularized tendon, which allows, when needed, the complete reconstruction of the proximal radial-ulnar ligaments, or just the annular ligament, in order to stabilize the head of the radius within the elbow joint. Also, the technique doesn't require osteotomies or an osteosynthesis requiring another surgery to remove the synthesis materials.

## Introduction

**Fig. 1:**
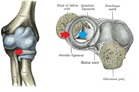
Anatomical elements ensuring the stability of the elbow: annular ligament (red arrow) and quadrate ligament (blue arrow) (after Grey)

The normal stability and mobility of the proximal radial-ulnar joint is ensured by the integrity of the annular and quadrate ligament (**Fig. 1**).

 The congenital radial head dislocation in young children requires the reconstruction of the radio-humero-ulnar joint to avoid the limitation of the flexion and supination, depending on the type of the displacement, and to elude the severe complications that may culminate with the paralysis of radial or ulnar nerves.

 Due to the fact that the ligament plasty with fascia lata or by the Bell-Tawse procedure modified by Lloyd Roberts does not avoid the risk of resorbtion of the neoligament or may induce an excessive limitation of the pronation and supination, we have practiced ligament plasty with ECRL in 2 cases of radial head displacement, one being congenital and the other posttraumatic.

 We considered the opinion of the advocates of osteotomy of the ulna which claim that reconstruction of the annular ligament alone cannot maintain the reduction of the radial head, but in most series there was a high rate of complications due to ulnar osteotomy [**1**].

 Traumatic radial head dislocation is most often associated with the fracture of the ulna, a lesion known as a Monteggia-Stanciulescu fracture, but can also occur in isolation.

## Materials and Method

 Surgical technique

 An incision is made on the dorsal side of the elbow, starting on the lateral side of the triceps tendon, reaching the cutaneous dimple corresponding to the radial head and heading laterally along the medial edge of the ECRL, ending at the tendon-muscle junction. After dissecting the subcutaneous tissue, the brachial fascia, articular capsule and the insertion of the aponeurosis of the epicondylian muscles are exposed.

 The antebrachial fascia is exposed and incised to reveal the extensor carpi radialis longus (ECRL) and the extensor carpi radialis brevis (ECRB) muscles. In addition, the ECRB and ECRL tendons are identified and isolated in the distal extremity.

**Fig. 2:**
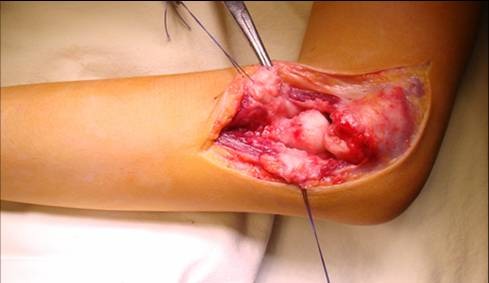
The radial head is ventrally dislocated

 The articular capsule situated above the epicondylian muscles is cut to reveal the humeral condyle and the head of the radius, dislocated ventrally, laterally or dorsally (**Fig. 2**). 

 The exuberant tissue that has grown in the radial notch of the ulna is excised, after checking for the presence of any remaining length of annular ligament; this may be found in traumatic downward dislocations of the radial head. Reduction of the dislocation is attempted and, if reduction is possible, a tunnel is drilled from lateral to medial.

**Fig. 3:**
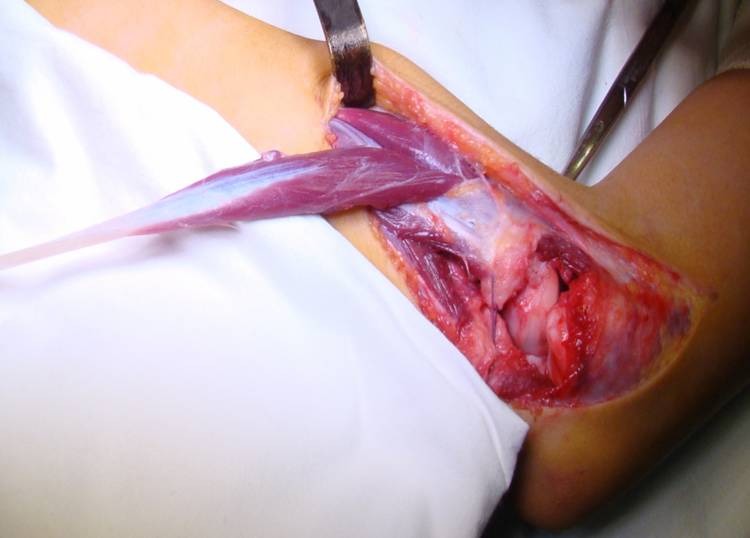
The ECRL tendon is exposed at the level of the elbow incision

 A 2 cm incision is made at the base of the second metacarpal and the insertion of the ECRL tendon is identified, isolated and cut. The tendon is pulled up to the distal end of the proximal incision (**Fig. 3**).

 The last 2 cm of the free end of the tendon are tied with atraumatic suture thread using Cuneo type sutures.

**Fig. 4:**
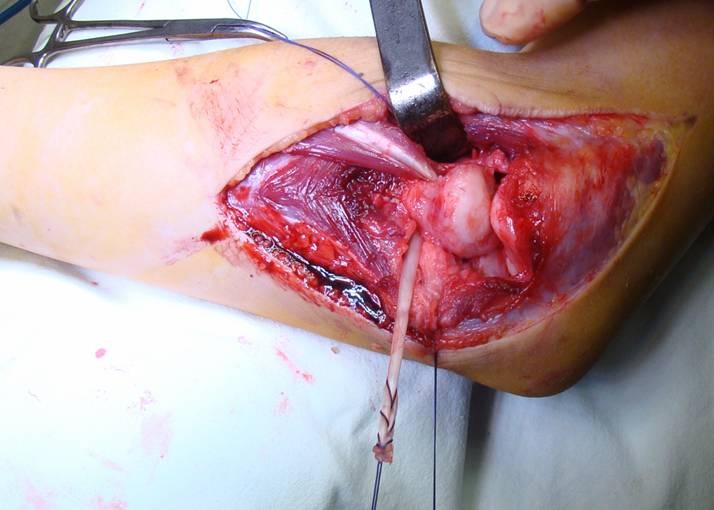
The tendon is passed through the bone tunnel in the radial neck

**Fig. 5:**
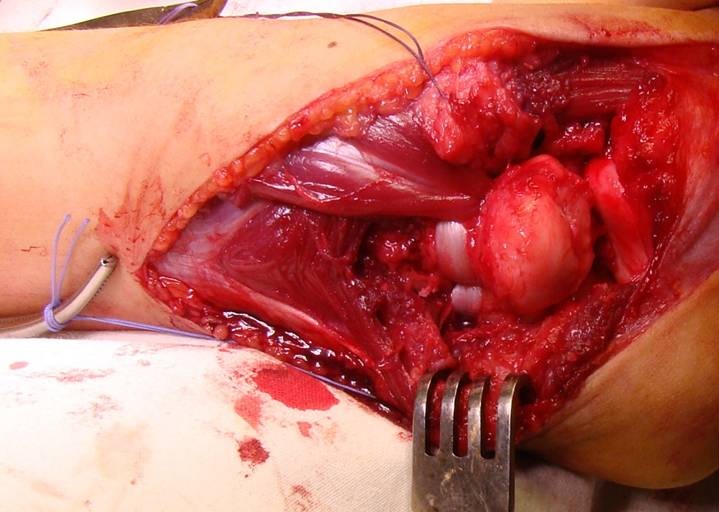
Reconstructed annular ligament, fixed in the strap by the muscle belly of the ECRL. Radial head in a normal position relative to the ulna and humerus

 The ECRL tendon is passed through a gap made through the ERCB below the tendon-muscle junction. The proximal end of the gap is closed with 1 - 2 suture points. The tendon is passed through the radial neck tunnel (**Fig. 4**), then around the radial neck, under the muscular part, either posteriorly (**Fig. 5**) or anteriorly, depending on the type of dislocation. The tendon is then passed under the strap in order to prevent recurrent dislocation and fixed to the ulna at the insertion points of the radial ring using an interference screw. 

 Reconstruction of the annular ligament and the check for stability are done with the elbow extended. After the distal end of the tendon is permanently fixed in the ulnar tunnel using the interference screw, the proximal end of the radius should be located normally in relationship with the humeral condyle and the ulna. After this, when the elbow is flexed during surgery, the ligament construct should relax and the head of the radius should move slightly in the direction of the original dislocation. Lateral X-rays of the flexed elbow show this minor dislocation in the first 3–4 months after surgery, then the subluxation in flexion should disappear. This technical artifice is intended to prevent the progressive decrease of pronation and supination range of motion. 

 Final screw fixation to the bone must insure stability while testing flexion and extension, and allow normal pronation and supination.

 The subcutaneous layers are closed anatomically and the skin is sutured.

 A long-arm splint is applied, with the elbow at 90°, and is kept for 2 - 3 weeks, depending on the age of the child. This study analyses two cases of radial head dislocation, one congenital and one traumatic

 Case 1

**Fig. 6:**
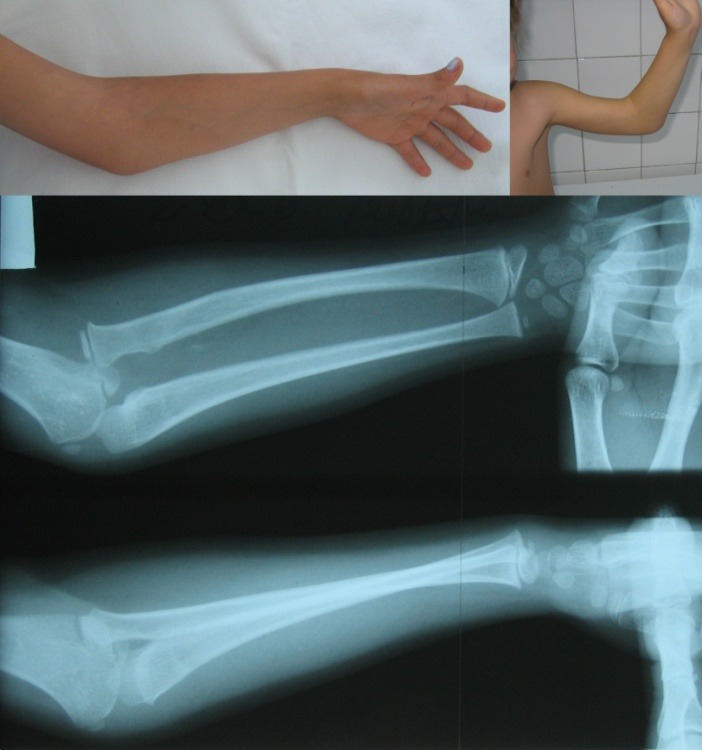
Case 1: Preoperative clinical and radiological appearance

 A 6-year-old female patient, J.L.C., sought medical attention in our clinic in 2006, when she was diagnosed with anterior congenital dislocation of the left radial head. Clinically, elbow flexion was limited to 90°, extension by 20° and supination was limited to 20–30° (**Fig. 6**).

**Fig. 7:**
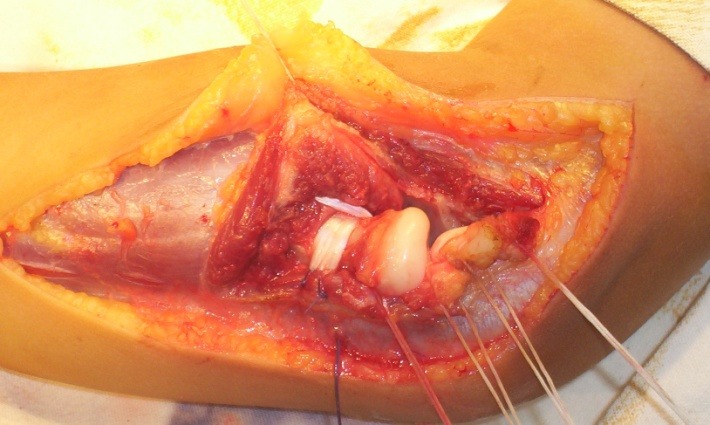
Case 1: Proximal radial-ulnar ligament plasty, variant 2

**Fig. 8:**
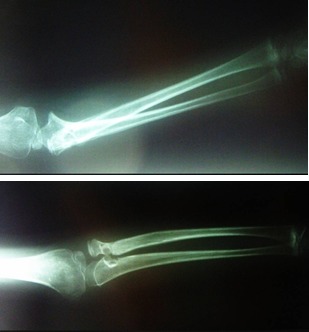
Case 1: X-rays two months after surgery. Images reflect normal position of radial head relative to the ulna and the humeral condyle

**Fig. 9:**
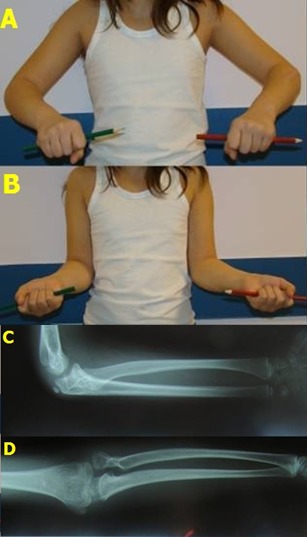
Clinical and radiologic check-up at two years after surgery. A. Maximum pronation; B. Maximum supination; C. Lateral X-ray – radial head in anatomic position; D. Anterior-posterior X-ray – normal relationship between the head of the radius and the humeral condyle.

 In May 2007 the patient underwent surgical treatment by proximal radial-ulnar ligament plasty, Burnei’s technique variant 2 (**Fig. 7**). Postoperatively, evolution was favorable (**Fig. 8**), with active elbow motion within normal limits at two years follow-up (**Fig. 9**).

 Case 2

 An 11-year-old male patient, D.Y., had suffered, in October 2008, a traumatic event affecting the left forearm, and treated by casting. Three months later, swelling on the ventral side of the elbow and limited flexion were noticed. An elbow X-ray diagnosed posttraumatic dislocation of the left radial head. In January 2009 the patient underwent annular ligament reconstruction, using the common extensor tendon, and fixation of the radial head using transcondylian Kirschner wires.

**Fig. 10:**
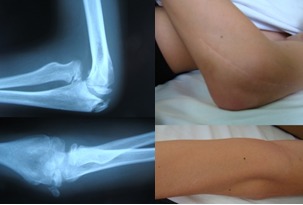
Clinical and radiologic appearance four years after the first surgery and before proximal radial-ulnar ligament plasty

**Fig. 11:**
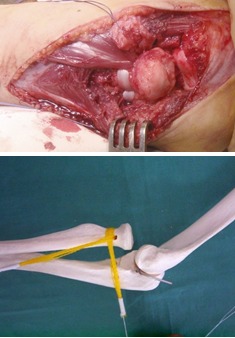
Ligament plasty for the anterior dislocation of the radial head, second variant of Burnei’s technique

**Fig. 12:**
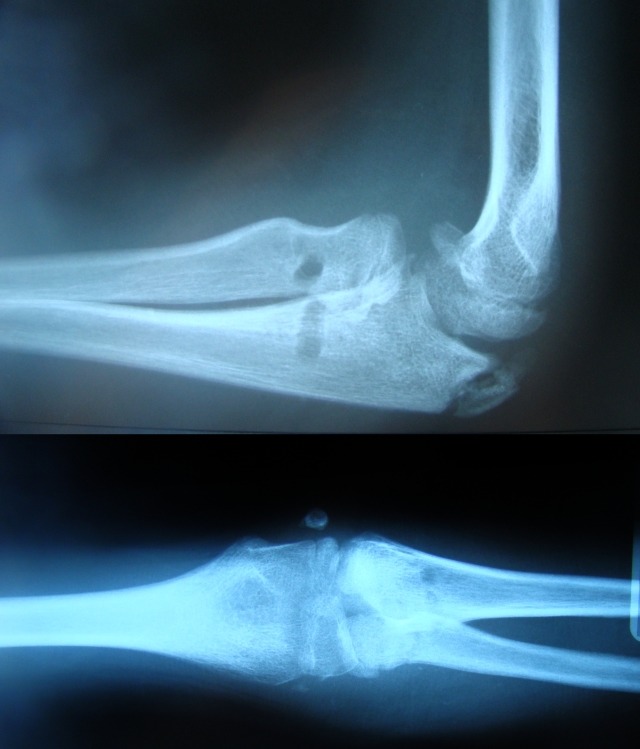
Radiologic aspect 30 days after surgery. There are two radiolucent areas corresponding to the tunnel through the radial neck and the point where the ulnar interference screw is inserted. The lateral X-ray, with the elbow flexed, shows a slight dislocation of the radial head in relation to the ulna; this position is required by the technique in order to avoid the constriction of the radial neck and subsequent progressive limitation of pronation and supination. The anterior-posterior X-ray, with the elbow extended, shows a normal placement of the radial head in relation with the ulna and the humeral condyle.

 Five months after this intervention, the symptoms recur and the control X-rays show the redislocation of the radial head (**Fig. 10**), however the parents momentarily decide against continued treatment. Four years after recurrence, surgery is again performed, using Burnei’s technique for proximal radial-ulnar ligament plasty, variant 2 (**Fig. 11**). Postoperative evolution at one and two months is favorable (**Fig. 12and13**).

**Fig. 13:**
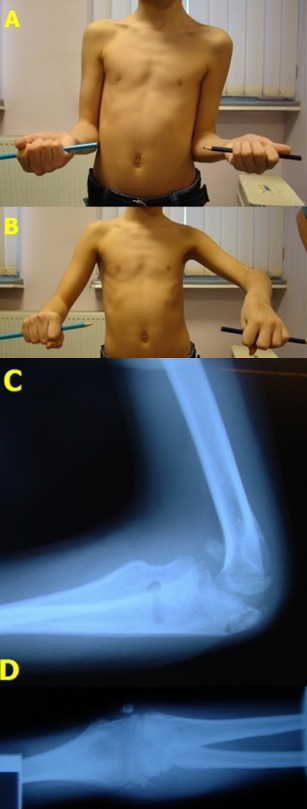
Clinical and radiological 2 months follow-up. A. Normal supination. B. 20 degrees limited left antebrachial pronation. C. Lateral X-rays showing the limiting of radial head relaxation. D. Normal positioning of the radial head.

## Results

 During both surgeries, the radial head was reduced and stabilized in relation with the humerus and ulna. The follow-up period for the congenital radial head dislocation is two years. At two years follow-up, elbow flexion and extension, and also forearm pronation and supination were normal. Anterior-posterior and lateral elbow X-rays showed a normally situated radial head, in relation with the humeral condyle.

 The patient with traumatic dislocation of the radial head was followed-up postoperatively for two months. At the last check-up, elbow flexion was normal, pronation was limited with 20 degrees and supination normal. Elbow X-rays displayed a slight displacement of the head of the radius with regard to the ulna; this positioning had been necessary to avoid the constriction of the neck of the radius and subsequent progressive limitation of pronation and supination. On the anterior-posterior X-ray with the elbow in extension, the radial head was normally positioned in relation with the ulna and humeral condyle. 2 month postoperative, the displacement reduced significantly when flexing the elbow.

 Complications

 In the case of the traumatic dislocation, the tension created by the reduction of the anteriorly displaced radial head caused blisters to appear on the arm and forearm. After debridement, the blistered skin healed without further intervention.

## Discussions

History

**Fig. 14:**
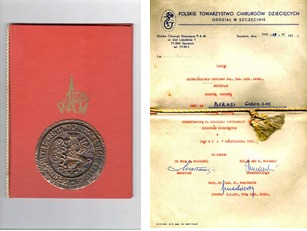
Polish Academy of Pediatric Surgery Award, won by the authors of the first paper on the surgical treatment of radial head dislocation by ligament plasty using the tendon of the ECRL

 Proximal radial-ulnar ligament plasty using the extensor carpi radialis longus (ECRL) tendon was first performed in 1986 by G. Burnei at Mangalia Municipal Hospital in Romania. In September 1989, the scientific paper “Ligamentoplastia radioulnară proximala în luxația congenitală a capului radial – procedeu original" [Proximal Radial-ulnar Ligament Plasty for the Congenital Dislocation of the Radial Head – Original Procedure] (G. Burnei, A. Faur, C. Dumitrescu, L. Mârza) was presented at the Iași International Congress of Pediatric Surgery and Orthopedics [**2**]. The paper showed the results obtained on 4 cases (3 congenital dislocations, aged 1-3, and one traumatic dislocation, aged 6), and demonstrated the technique's advantages. The congress organizers were apprehensive about the term "original procedure", and the congress documentation contained the simplified title "Proximal Radial-ulnar Ligament Plasty for the Congenital Dislocation of the Radial Head"; the presenter however mentioned explicitly that the procedure was original. The paper received the Polish Academy of Pediatric Surgery Award, bestowed by Prof. Dr. C. Z. Markiewicz (**Fig. 14**).

The clinical experience gathered along the years on proximal radial-ulnar ligament plasty has been synthesized in several scientific papers, presented at: Orthopedics Jubilee Meeting, 1-3 June 1994, Craiova, Romania [**3**]; The 11th SOROT National Congress of Orthopedics and Traumatology, October 19 - 21 2005, Bucharest, Romania [**4**]; The 29th Annual Congress of the European Pediatric Orthopedic Society (EPOS), April 7-10 2010, Zagreb, Croatia [**5**].

 The latest paper, presented at the 29th Annual Congress of the EPOS by the authors, G. Burnei, S. Gavriliu, I. Georgescu, C. Vlad and L. Hurmuz, is based on 19 cases of radial head dislocation, 8 congenital and 11 traumatic, on which 20 surgeries were performed. The patients were aged between 3 and 14, on average 6.4 years old, at the time of surgery. The results were very good for 13 patients (14 surgeries - one patient was operated bilaterally), good for 4 patients and mediocre for 2 patients. 17 patients had less than 30° of supination loss. The most frequent long-term complication recorded by the authors was radio-humeral arthrosis, diagnosed in 3 patients.

 Comments

 A number of other authors have described surgical techniques for the reestablishment of normal relationships between the head of the radius and the radial notch of the ulna on the one hand, and the head of the radius and the capitellum on the other. Some of these are: annular ligament reconstruction using a tendon bundle from the triceps brachii (Bell Tawse 1965; Boyd and Boals 1969; Lloyd-Roberts and Bucknill 1977; Hurst and Dubrow 1983; Seel and Peterson 1999), a portion of the antebrachial fascia (Bell Tawse 1965; Boyd and Boals 1969), the palmaris longus tendon (Lloyd-Roberts and Bucknill 1977); the Varna procedure, using surgical thread to reconstruct the annular ligament; ulnar lengthening and angulation osteotomy (Lord and Roy-Camille 1962; Nishio et al. 1965; Lloyd-Roberts and Bucknill 1977); radial osteotomy (Yamamoto et al. 1976) and not least ligament reconstruction with ulnar osteotomy (Lloyd-Roberts and Bucknill 1977; Fowles et al. 1983).

 Boyd and Boals stated that the use of a postero-lateral incision the radial head is defined and the capsular block exposed and removed. The radial head is then easily reducible without the need to divide the ulna. A new annular ligament is made by turning down a slip of the triceps tendon, leaving it attached to the ulna, and passing it round the neck of the radius from behind forward and securing it through a drill hole in the ulna [**6**].

 Bell Tawse remarked that reduction, possible with the arm flexed, was impossible with the arm extended because of an apparent disparity in the length of the radius and ulna. It was reduced in the flexed position and held in place by making a new annular ligament [**7**]. Bell Tawse (1965) used a central bundle from the triceps brachii tendon, which was lowered, passed through a tunnel through the olecranon, wrapped around the radial neck and then fixed to the lateral side of the ulna.

 Lloyd-Roberts and Bucknill used, in some cases, the palmaris longus tendon in this surgical technique. Notably, they introduced two modifications to the Bell Tawse technique: they used a lateral triceps brachii tendon bundle that they lowered to the level of the radial neck in order to reconstruct the annular ligament, and they inserted a transcondylar K wire that stabilizes the radial head, as demonstrated by Lambrinudi (1939). Although satisfactory results were obtained with both methods, he preferred reconstruction with the triceps tendon, for it confines the operation to one surgical field, and the preservation of the normal ulnar attachment inspires confidence in the viability of the refashioned ligament [**8**].

 Hurst and Dubrow also lowered a central bundle from the triceps brachii tendon, but modified the Bell Tawse technique: further, it is important to strip the tendon of the proximal olecranon with a 2- to 3-cm segment of dorsal periosteum so that the attachment site of the newly reconstructed ligament will be parallel to the radial neck and not proximal to it. This alignment more closely approximates the normal anatomy of the annular ligament [**9**], unlike fixation to the olecranon.

 Seel and Peterson described a method in which they drilled two holes in the proximal ulna, at the level of the origins of the annular ligament, in order to repair the original ligament or to reconstruct it using a tendon bundle from the triceps brachii, stabilized with two interference screws. They stated that using two points of fixation to the ulna greatly increase the stability of the radial head, compared to Bell Tawse's technique. It secures the radial head in its normal position from any dislocated position. It also allows for osteotomy of any accompanying deformity of the ulna or radius [**10**].

 T. Hirayama et al. (1987) were reticent about annular ligament reconstruction, considering it inadequate for the anatomical contention of the radial head and that re-dislocation would follow. In addition, narrowing and restriction of rotation of the radial neck may be caused by excessive tension in the reconstructed ligament; in children, inhibition of growth of the radial head by the reconstructed ligament must also be considered [**11**]. Regardless, most authors employed surgical techniques that included the reconstruction of the annular ligament, with or without an ulnar osteotomy.

 Ulnar osteotomy, with or without annular ligament reconstruction, had been seen as an effective method to preserve the reduction of the radial head, but in many cases a high rate of postoperative complications was reported. Oner and Diepstraten (1984) published a study in which 7 patients with traumatic dislocation of the radial head were operated using Lloyd-Roberts and Bucknill`s technique of annular ligament reconstruction, without osteotomy. Four patients had a good outcome, two had a satisfactory outcome, exhibiting recurrent dislocation, and one child developed synostosis because of the lack of the use of a tourniquet. Their results confirm that open reduction with ligament reconstruction by a triceps tendon slip is a reliable operation for anterior dislocations. In this group they had only one poor result, because of the development of a synostosis. Osteotomy is not without complications. Recently, Hirayama et al reported two broken bone plates in nine osteotomies and residual subluxation in one. Verneret et al. (1989) described 11 good results in 14 patients but two had to be reoperated and two suffered neurological complications. Bouyala et al. (1988) reported five cases of delayed union and loosening of the plate in 15 cases of post-traumatic, paralytic and congenital dislocations. We think that osteotomy is justified only in those cases in which stable reduction is not possible without it, or when the deformity may cause later subluxation such as occurs in antero-lateral dislocation [**11**].

**Fig. 15:**
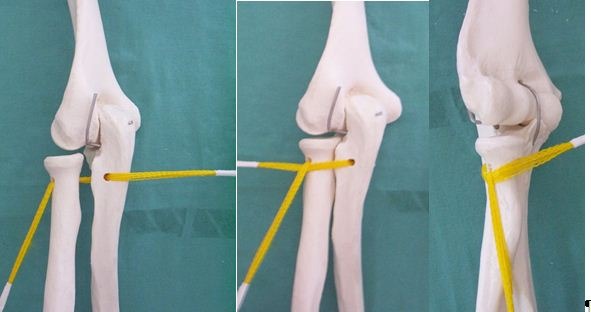
Technique for the reconstruction of both ligaments

 The first series of proximal radial-ulnar joint stabilizations done by the author included four cases and used the technical variant involving the reconstruction of the quadrate and annular ligaments (**Fig. 15**).

 In evolution, this variant has the disadvantage of progressive limitation of pronation-supination, down to approximately 30°. Subsequently, other technical variants were used, depending on the type of dislocation.

**Fig. 16:**
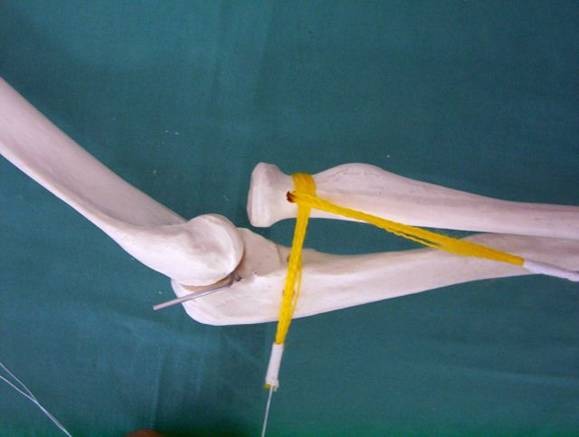
Ligament plasty technique for anterior dislocation

 Variant 2 is used in anterior dislocations. The tendon is passed through a tunnel drilled in the radial neck, wrapped around the anterior half of the circumference, under the strap, and fixed at the level of the posterior insertion of the annular ligament (**Fig. 16**).

**Fig. 17:**
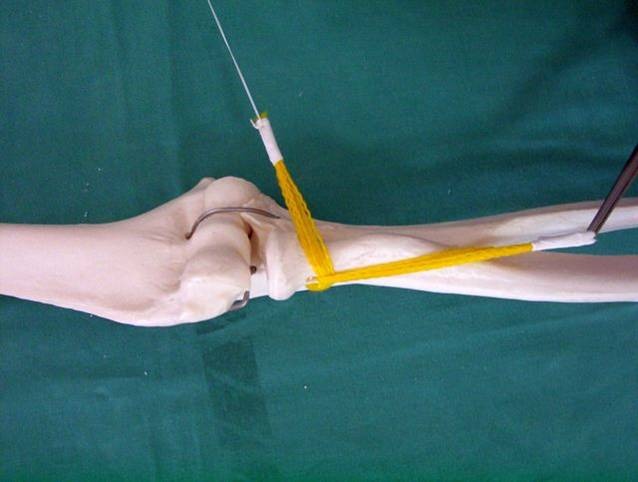
Ligament plasty technique for posterior dislocation

Variant 3 is used in posterior dislocations. The tendon is passed around the posterior half of the circumference and fixed with an interference screw at the anterior insertion of the annular ligament (**Fig. 17**).

**Fig. 18:**
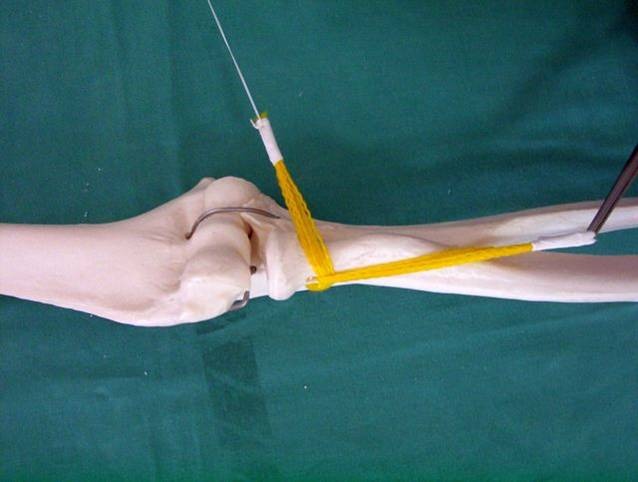
Ligament plasty technique for lateral dislocation; it may be also used for the other forms

In Variant 4, the tendon is passed through a tunnel in the radial neck, from medial to lateral, divided in two equal strips, which are passed one anterior and one posterior and then fixed to the insertion points of the annular ligament. This fourth variant can be used in all forms of dislocation of the radial head (**Fig. 18**).

 Variants 2, 3 and 4 are advantageous in that the radial neck is only partially circumscribed, which avoids constriction of the radial neck during growth. 

## Conclusions

The Burnei procedure is an alternative for the treatment of radial head dislocation and is advantageous because of the use of a sturdy, well vascularized tendon, which allows, when needed, the complete reconstruction of the proximal radial-ulnar ligaments, or just the annular ligament, in order to stabilize the head of the radius within the elbow joint.
